# Modulation of gut microbiota, up-regulation of ZO-1, and promotion of metabolism as therapeutic mechanisms of indole-3-carbinol against obesity in mice

**DOI:** 10.3389/fphar.2024.1499142

**Published:** 2025-01-03

**Authors:** XuWen Mao, Guliruoyi Paerhati, Yuche Wu, Lu Feng Cheng

**Affiliations:** ^1^ College of Pharmacy, Xinjiang Key Laboratory of Biopharmaceuticals and Medical Devices, Xinjiang Medical University, Ürümqi, China; ^2^ Xinjiang Technical Institute of Physics and Chemistry, Chinese Academy of Sciences (CAS), Ürümqi, China

**Keywords:** indole-3-carbinol, gut microantha biota, obesity, serum metabolomics, ZO-1

## Abstract

**Background:**

Indole-3-carbinol (I3C) is a compound derived from Cruciferous vegetables. We aim to ascertain whether I3C mediates the relations between mouse gut microbiota, intestinal barrier function, and metabolism to treat obesity in mice.

**Methods:**

The experimental analyses focused on the changes in lipid distribution, inflammatory cytokines, glucose tolerance, gut microbiota composition, and serum metabolomics of 60 C57BL/6N mice.

**Results:**

The experimental results demonstrated that I3C reduced body weight, hepatic steatosis, and systemic inflammation and improved insulin resistance in mice on a high-fat diet (HFD). Furthermore, I3C remarkably enhanced the enrichment of probiotics *Akkermansia* and *Ligilactobacillus* as well as SCFA-producing bacteria (*Eubacterium*, *Lactococcus*, and *Coprococcus*), while reducing the abundance of *Eisenbergiella* and *Rikenellaceae_RC9_gut_group*. Also, I3C notably up-regulated the levels of Claudin4, Occludin, and ZO-1 proteins and modulated the metabolism of argininosuccinic acid and galactose.

**Conclusion:**

The aforementioned findings suggest that I3C exerts a significant anti-obesity effect in mice by regulating abnormal gut microbiome, enhancing intestinal barrier function, and improving metabolic disorders.

## 1 Introduction

At present, global residents tend to have a dietary habit of high-fat and low-dietary fiber intake. Vegetable deficiency in the daily diet leads to masked obesity in the body, such as low-grade inflammation, insulin resistance, and abnormal lipid metabolism (Finucane et al., 2011; Mihaylova et al., 2014). It is expected that by 2030, the global prevalence of male obesity will reach 18%, and that of female obesity will reach 21% (Sadali et al., 2023).

Indole-3-carbinol (I3C) is sourced from Cruciferous vegetables. Indole glucosinolates in cruciferous vegetables are cleaved by myrosinase during the digestion of vegetables, producing isothiocyanates and I3C (Bjeldanes et al., 1991; Andersson et al., 2002). I3C can strengthen intestinal barrier integrity and elevate the resistance of mice to dextran sulfate sodium-evoked colitis, which is beneficial to the maintenance of intestinal health (Kim and Milner, 2005). Cruciferous vegetables also contain other active substances, including anti-cancer and antioxidant glucosinolates (Glucosinolates, GSL), indole glucosinolates that prevent inflammatory lesions, and sulforaphane (SFN) that regulates inflammatory responses (Johnson, 2018; Ağagündüz et al., 2022). I3C activates the Aryl hydrocarbon receptor (AHR) protein for the prevention of colitis and colon cancer (Furumatsu et al., 2011). AHR-deficient mice are prone to develop intestinal inflammation, which in turn progresses into colon cancer; An I3C-rich diet normalizes intestinal stem cell proliferation, expedites their differentiation into intestinal epithelial cells and goblet cells, augments the expression of tight junction proteins, increases intestinal barrier integrity, and reduces the incidences of colitis and colon cancer (Hao et al., 2012; Jiang et al., 2013; Hubbard et al., 2015). Since I3C can repair the intestinal mucosal barrier, its positive role in the prevention and treatment of obesity and metabolic syndromes attracts more and more attention. Only about 5%–10% of indoles in vegetables can be absorbed in the small intestine. However, the remaining indoles are enriched in the large intestine, which can serve as energy substrates for some probiotics, facilitate the growth of probiotics, and also restrain the growth of pathogenic bacteria (Kleman et al., 1994; Kim and Milner, 2005; Kiss et al., 2011; Lee et al., 2011; Jiang et al., 2013). Therefore, the mediating effect of indole compounds on gut microbiota is considered to be a key modulator of health effects.

The dysbiosis of the gut microbiome is correlated with a wide range of diseases, such as neurological disorders, cardiovascular diseases, gastrointestinal diseases, and even cancer. Particularly, the diversity and key core species of the gut microbiota in obese and type 2 diabetes patients are capable of participating in the regulation of metabolism, immunity of the host. The gut microbiome might be employed as a component of precision medical approaches in the future for disease diagnosis, prognosis, and treatment intervention. Regulating the gut dysbiosis holds great significance for maintaining overall health and preventing and treating various diseases. By supplementing microecological agents, using antibiotics rationally, and considering fecal microbiota transplantation when necessary, the gut microbiota can be effectively regulated to promote health.

An imbalance of gut microbiota can cause an increase in Gram-negative gut pathogenic bacteria (such as *Escherichia coli* and *Enterococcus*), leading to increased lipopolysaccharides (LPS), exacerbated intestinal epithelial inflammation, and impaired intestinal barrier. Increased absorption of LPS into the blood circulation contributes to endotoxemia and triggers inflammatory reactions in the body (Zhao, 2013; Brahe et al., 2016). The LPS levels of obese mice on a high-fat diet (HFD) are 2–3 times higher than healthy mice, with chronic low-grade inflammation occurring in obese mice (Suárez-Zamorano et al., 2015). Supplementation of probiotics or dietary supplements can increase gut probiotic levels, restore the structure of gut microbiota, and alter the release of metabolites like short-chain fatty acids (SCFAs), thereby ameliorating obesity (Qin et al., 2010; Gomes et al., 2018). Therefore, regulating the gut microbiota is a therapeutic target for the development of anti-obesity drugs. I3C, as the most readily available indole compound, has shown great potential in obesity management. However, the impact of I3C on obesity and its associated metabolic syndrome are rarely investigated. It deserves further investigation on whether I3C can play a beneficial therapeutic role in obesity by regulating the microbiota, promoting intestinal barrier integrity, reducing inflammation, and controlling blood glucose and lipid levels.

This study utilized high-throughput sequencing to demonstrate the mediating role of I3C in the gut microbiota of mice, explore the changes in gut microbiota in mice receiving a normal diet or HFD, the relationships between changes in gut microbiota and changes in parameters such as serum metabolites, blood lipids, blood glucose, and cytokines induced by I3C therapy. Meanwhile, this study probed into the possible mechanisms of the initiation and development of obesity as well as I3C therapy, providing a theoretical and experimental foundation for the clinical treatment of obesity.

## 2 Materials and methods

### 2.1 Experimental drugs

I3C was procured from Shanghai yuanye Bio-Technology Co., Ltd. (S49927); Orlistat enteric tablets were commercially available from Zhejiang Hisun Pharmaceutical Co., Ltd. (H20100089); Metformin was supplied by ChongQing KeRui NanHai Pharmaceuticals Ltd. (691,504).

### 2.2 Animals and treatments

Sixty 5-week-old male C57BL/6N mice weighting 18–22 g (Beijing Vital River Laboratory Animal Technology Co., Ltd.) were kept in the standard laboratory (25°C ± 2*°C*, 12-h light/12-h dark cycle) without food and water restriction. All experiments concerning animals were ratified by the Ethics Committee of Xinjiang Medical University (Ethic No.: IACUC-20211118–02).

Following a one-week acclimation period, the mice were assigned at randomization into 6 groups with 10 mice/group: NCD (mice on a normal diet), NCD-I3C (mice on a normal diet administrated with I3C), HFD (mice on an HFD), HFD-I3C (mice on an HFD administrated with I3C), HFD-Orlistat (HFD-O, mice on an HFD administrated with Orlistat), and HFD-Metformin (HFD-M, mice on an HFD administrated with Metformin) groups. The normal diet contained 10% kcal from fat (supplied by the Animal Center of Xinjiang Medical University), while the HFD comprised 60% kcal from fat (Beijing Vital River, D12492).

Mice in the NCD and HFD groups were administered intragastrically with an equivalent amount of normal saline. Mice in the NCD-I3C and HFD-I3C groups were orally administered with I3C, 40 mg/kg/day. Mice in the HFD-Orlistat were orally given Orlistat, 60 mg/kg/day, and the mice in the HFD-Metformin groups were orally given Metformin, 120 mg/kg/day. The weight and food intake of all mice were measured once a week. Fresh fecal samples from all mice were harvested and then preserved in a −80*°C* freezer. After intraperitoneal injection of pentobarbital sodium (35 mg/kg) for anesthesia, mouse eyeball vein blood was extracted and placed in a heparin-containing blood collection tube, followed by 15-min centrifugation (4*°C*, 1000 g). The plasma was separated and preserved at −80*°C*. The liver tissues and colon tissues were harvested, one portion of which was fixed in 4% paraformaldehyde for 24 h, while the other portion was placed in a −80*°C* freezer for later use.

### 2.3 Hematoxylin and eosin staining

Liver and colon tissues fixed in 4% paraformaldehyde were sheared, followed by graded dehydration in 50%, 75%, and 95% ethanol. The tissues were hyalinized after 2 h of immersion in xylene. After paraffin embedding, the tissues were sliced into 5-µm sections and then hydrated. The sections were dyed by hematoxylin-eosin for 5 min, rinsed in water for 10 min, and then washed in distilled water. Following 2-min dehydration in 95% ethanol, the sections were rinsed with distilled water 2 times. Next, the sections were hyalinized in xylene for 5 min, followed by 2 rinses in distilled water again. These sections were sealed with neutral resin. Ultimately, the pathological and morphological changes of mouse liver and colon tissues were visualized under the microscope and photographed by the electron microscope (Aximujiang et al., 2022).

### 2.4 Body fat composition analysis

Body fat compositions (fat, muscle, and body fluid contents) in all mice were analyzed on a small animal nuclear magnetic resonance (NMR) (Nanjing Xinfeida Oetech. co.,LTD., LF50) body fat analyzer.

### 2.5 Analysis of the levels of blood lipid level and inflammation factor

The ELISA kits were adopted for the measurements blood lipid level of T-CHO (Nanjing Jiancheng Bioengineering Institute, A111-1–1), TG (Nanjing Jiancheng Bioengineering Institute, A110-1–1), HDL-C (Nanjing Jiancheng Bioengineering Institute, A112-1–1), LDL-C (Nanjing Jiancheng Bioengineering Institute, A113-1–1).

Inflammation factor in the serum were measured via ELISA kits, IL-6 (Quanzhou Ruixin Biotechnology Co., Ltd., RX203049M), IL-1β (Shanghai Jianglai Biotechnology Co., Ltd., JL18442), CXCL1 (Shanghai Jianglai Biotechnology Co., Ltd., JL20150), MPO (Shanghai Jianglai Biotechnology Co., Ltd., JL10367), IFN-γ (Shanghai Jianglai Biotechnology Co., Ltd., JL10967) and TNF-α (Quanzhou Ruixin Biotechnology Co., Ltd., RX202412M).

ALT and AST activities in the serum were measured via an ALT assay kit (Shanghai Jianglai Biotechnology Co., Ltd., JL12668) and a AST assay kit (Shanghai Jianglai Biotechnology Co., Ltd., JL13992).

### 2.6 Immunohistochemical staining

Colon tissue sections were subjected to dewaxing, gradient ethanol dehydration, antigen retrieval, and PBS rinses. Sections were incubated with 10% goat serum, primary antibody (overnight, 4°C), and secondary antibody (37°C, 30 min). After DAB staining, the cell nucleus was dyed by hematoxylin and sealed with neutral resin, followed by microscopic image acquisition. The positive rate was analyzed based on the acquired images using ImagePro Plus 6.0 software.

### 2.7 Assessment of glucose and insulin resistance

After 11 weeks of dietary intervention, mouse insulin and fasting blood glucose (FBG) levels were measured. ITT and OGTT experiments were simultaneously carried out. All mice were fasted for 8 h after the last lose, without water limitation. FBG levels were measured in mouse tail-tip blood samples by a glucometer. Then, each mouse was administered intragastrically with a glucose solution of 2 g/kg, and tail-tip blood samples were taken at 0, 30, 60, and 120 min, respectively, for the measurement of the blood glucose levels. In the 12th week of the experiment, mice were subcutaneously injected with conventional insulin (0.5 U/kg). The 0-, 40-, 90-, and 120-min tail-tip blood glucose levels were again measured on the glucometer. The changes in blood glucose levels from t = 0 to t = 120 were analyzed, yielding the total area under the curve (AUC). An insulin ELISA kit was utilized for insulin level detection. The insulin resistance (HOMA-IR) was computed with the assistance of a steady-state model: fasting insulin (µUI/mL) × FBG (mM)/22.5.

### 2.8 DNA extraction and high-throughput sequencing

DNA was extracted from fecal samples by the cetyltrimethylammonium Bromide (CTAB) method, the quality of which was determined by agarose gel electrophoresis, followed by DNA quantitation on an ultraviolet spectrophotometer. The V3-V4 region of the bacterial 16S rRNA gene was amplified in the polymerase chain reaction (PCR) system (Bio-Rad, United States): amplification at 98*°C* for 30 s; subsequently, amplification at 98*°C* for 10 s, 54*°C* for 30 s, and 72*°C* for 45 s, 35 cycles in total; finally, extension at 72*°C* for 10 min using 341F 5′-CCTACGGGNGGCNGCAG-3′ and 805R 5′-GACTACHVGGGTATCTAATCC-3'. The PCR amplification reaction system (25 µL) comprised 12.5 µL Phusion Hot start flex 2×Master Mix, 2.5 µL Forward Primer, 2.5 µL Reverse Primer, and 50 ng Template DNA. Purification of PCR products was made by AMPure XT beads (BeckmanCoulterGenomics, Danvers, MA, United States), followed by quantitative detection by Qubit (Invitrogen, United States). After 2% agarose gel electrophoresis, products were harvested using the AMPure XT beads recovery kit. A bioanalyzer (2100Agilent, United States) and an Illumina library quantification kit (Kapa Biosciences, Woburn, MA, United States) were applied to assess the purified PCR products. Lastly, a NovaSeq 6000 sequencer was used for sequencing (Wang et al., 2020).

### 2.9 Bioinformatics analysis process

The double-ended data obtained after sequencing were split based on barcode information, and the connectors and barcode sequences were eliminated. The data were subsequently assembled and filtered. Length filtering and denoising were implemented using the QIIME2 software package and DADA2 plugin, generating Amplicon Sequence Variants (ASV) feature sequences and ASV abundance tables. Also, singletons ASVs were eliminated. The α-diversity and β-diversity were analyzed subsequently. α-diversity analysis can evaluate the diversity according to parameters like observed species, shannon, simpson, chao, goods coverage, and pielou e. β-diversity calculates four distances (weighted unifrac, unweighted unifrac, jaccard, bray curtis) for diversity assessment. With the ASV sequence files, the species were annotated on SILVA (Release 138, https://www.arbsilva.de/documentation/release138/) and NT-16S databases and their abundance in each sample was statistically analyzed on the strength of the ASV abundance table. In this analysis, a confidence threshold of 0.7 was used for annotations.

Based on the obtained species abundance information, a differential analysis was conducted. Different statistical methods were selected depending on the sample conditions: the Fisher’s exact test was applied for comparisons of samples without biological replicates; The Mann-Whitney *U* test and Kruskal–Wallis test were utilized for comparisons of samples with biological replicates between two groups and among multiple groups, respectively. Screening threshold: *P *< 0.05.

### 2.10 Blood sample preparation for metabolomics analysis

At first, 5 mL of abdominal aortic blood was extracted from mice and maintained at ambient temperature for 30 min. Following that, 15-min centrifugation was done at 3000 g and 4°C. The supernatant (serum) was packaged into an EP test tube. Then, 200 µL serum was mixed with 400 µL PBS in the EP tube before another 10-min centrifugation (10,000 g, 4°C). Next, 550 µL filtered serum was placed into a 5 mm NMR tube. Plasma ^1^H-NMR spectroscopy was performed with spin-echo pulse sequences on the Inova NMR spectrometer (Varian, United States). The water peak suppression was achieved using a pre-saturation method at 25°C, with a saturation time of 2 s, a sampling number of 32K, a scanning frequency of 128 times, and a spectral width of 1000Hz. The obtained ^1^H-NMR spectrum was subjected to phase and baseline corrections and processed using an exponential window function with a broadening factor of 0.5Hz. The width of each ^1^H-NMR spectrum (δ) was 9.0–0.5 ppm, and at the same time, the range for water signal removal was set as δH = 5.20–4.70 ppm.

### 2.11 Spectrum acquisition and metabolite quantification

Orthogonal partial least-squares-discriminant analysis (OPLS-DA) was implemented with the use of SIMCA-P11 software (Hong et al., 2022). The accuracy of OPLS-DA analysis results was assessed with the use of parameters such as R^2^X and Q^2^, wherein Q^2^ represents the validity of the statistical results. The multivariate statistical analysis of ^1^H-NMR spectral data involved principal component analysis, PLS-DA, and OPLS-DA. The OPLS-DA approach was employed to determine the variable importance in the projection (VIP) and determine the differences in metabolites between the two groups. VIP > 1 and P(Corr) > 0.3 were assigned as the identification criteria for differentially expressed metabolites.

### 2.12 Statistical analysis

All result analyses were realized with the application of GraphPad Prism version 9. Continuous variables were presented in the form of mean ± standard deviation, and categorical variables were described as percentages. Experimental results between the two groups were analyzed using unpaired *t*-tests. For the results among more than two groups, a one-way analysis of variance (ANOVA) was implemented, with the criteria being α = 0.05. Figures were plotted with the assistance of GraphPad Prism version 9.

## 3 Results

### 3.1 Effects of I3C on obesity parameters in HFD-fed mice

Relative to the NCD group, insignificant changes were detected in terms of obesity-associated parameters including body weight, weight gain, total fat content, white fat content, fat mass/body mass, lean mass/body mass, energy efficiency, body muscle content, and body fluid content in the NCD-I3C group. Except for body muscle content, other obesity-associated parameters mentioned above were elevated markedly in the HFD-fed mice (*P *< 0.05 or *P *< 0.01). The HFD-I3C, HFD-O, and HFD-M groups exhibited significant reductions in all the obesity-associated parameters mentioned above versus the HFD group (*P *< 0.05 or *P *< 0.01) (Figures 2A–I). Thus, I3C was suggested to be significantly effective for the treatment of HFD-associated obesity in mice.

**FIGURE 1:**
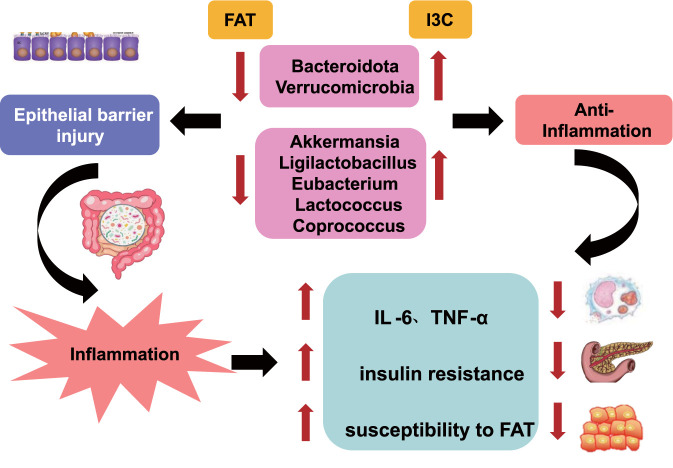
Possible mechanisms of FAT pathogenesis and I3C treatment for FAT.

**FIGURE 2:**
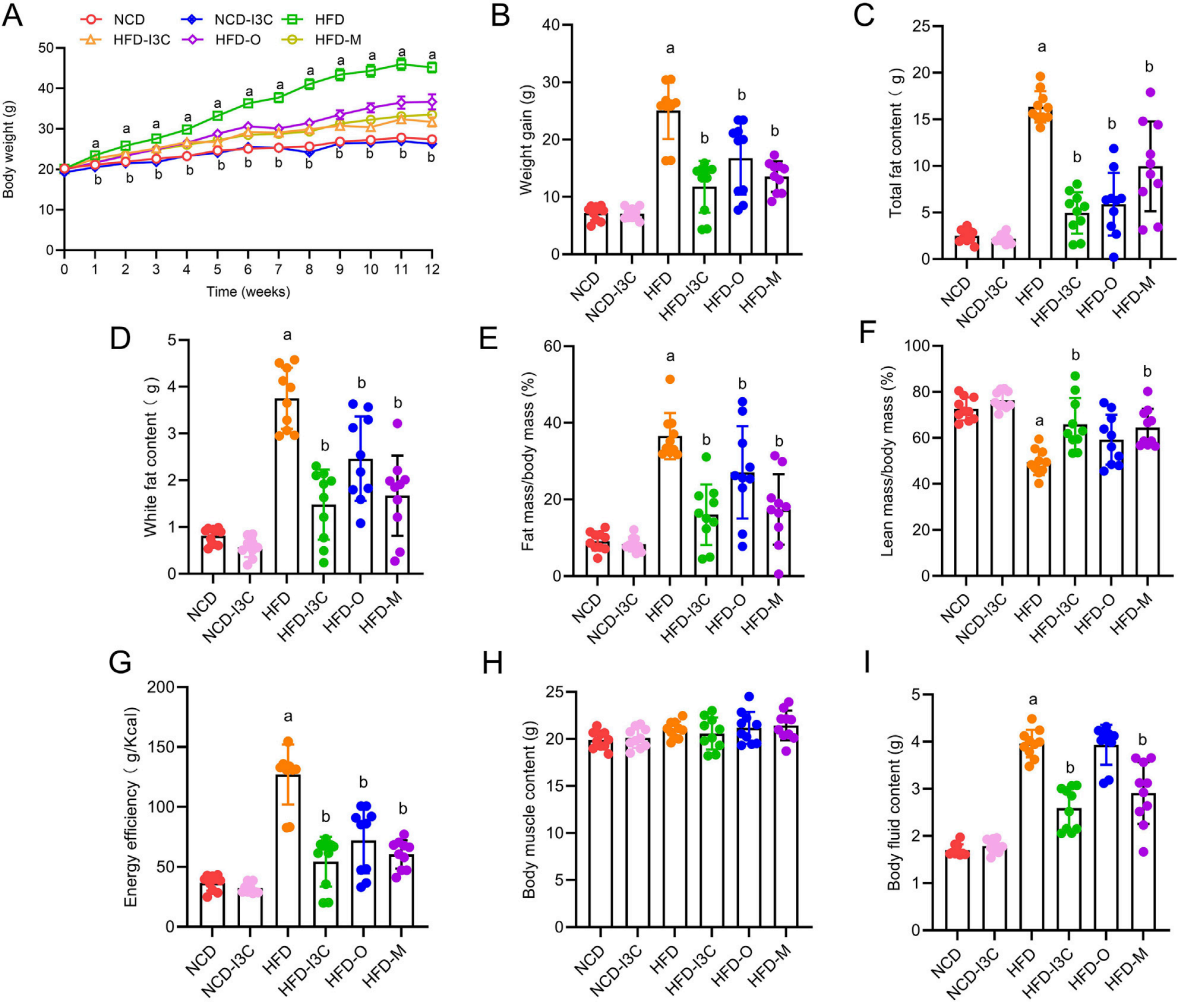
Effects of I3C (40 mg/kg) on treatment of obesity. **(A)** Body weight change of mice; **(B)** Weight gain of mice; **(C)** Total fat content of mice; **(D)** White fat content; **(E)** Fat mass/body mass; **(F)** Lean mass/body mass; **(G)** Energy efficiency of mice; **(H)** Muscle content of mice; **(I)** Body fluid content of mice. ^a^ *P* < 0.05, indicating comparison with NCD; ^b^ *P* < 0.05, indicating comparison with HFD. Data were expressed as mean ± SEM. (*n* = 10).

### 3.2 Impacts of I3C on blood glucose parameters in HFD-fed mice

**FIGURE 3:**
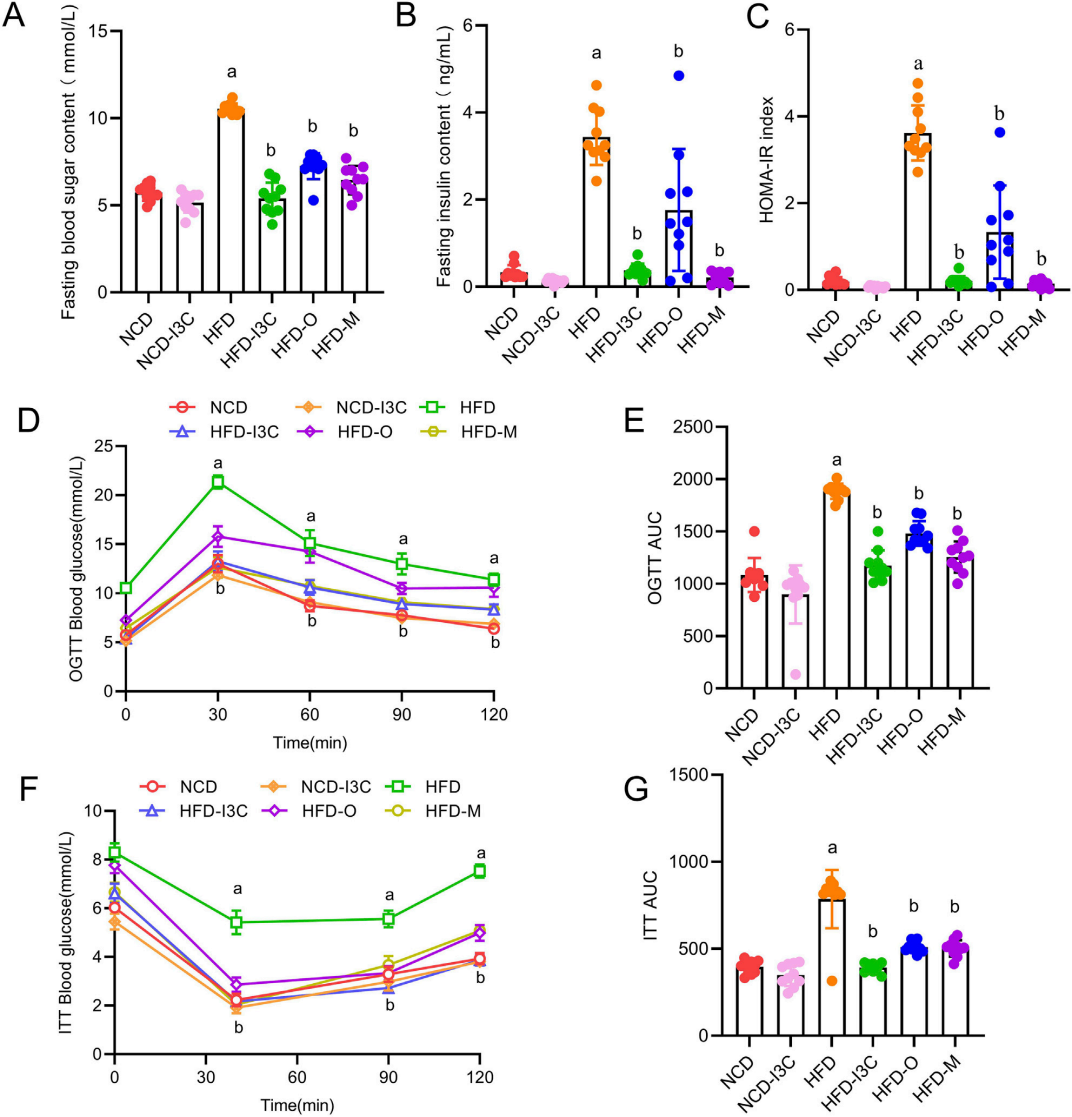
Treatment effects of I3C (40 mg/kg) on the development of insulin resistance induced by HFD feeding in mouse. **(A)** Fasting blood glucose; **(B)** Fasting insulin; **(C)** HOMA-IR; **(D)** Oral glucose tolerance test (OGTT); **(E)** OGTT AUC; **(F)** insulin tolerance test (ITT); **(G)** ITT AUC; ^a^ *P* < 0.05, indicating comparison with NCD; ^b^ *P* < 0.05, indicating comparison with HFD. Data were expressed as mean ± SEM. (*n* = 10).

FBG, insulin concentration, and HOMA-IR index showed no remarkable difference between NCD and NCD-I3C groups. However, these parameters were noticeably higher in the HFD group than in the NCD group (*P *< 0.05 or *P *< 0.01). The aforementioned parameters in the HFD-fed mice were markedly reduced in response to I3C, Orlistat, and Metformin treatment (*P *< 0.05 or *P* < 0.01) (Figures 3A–E). Furthermore, I3C exhibited a superior glucose-lowering effect to the positive control Orlistat but was less effective than the other positive control Metformin.

### 3.3 Influences of I3C on lipid and inflammation parameters in HFD-fed mice

**FIGURE 4:**
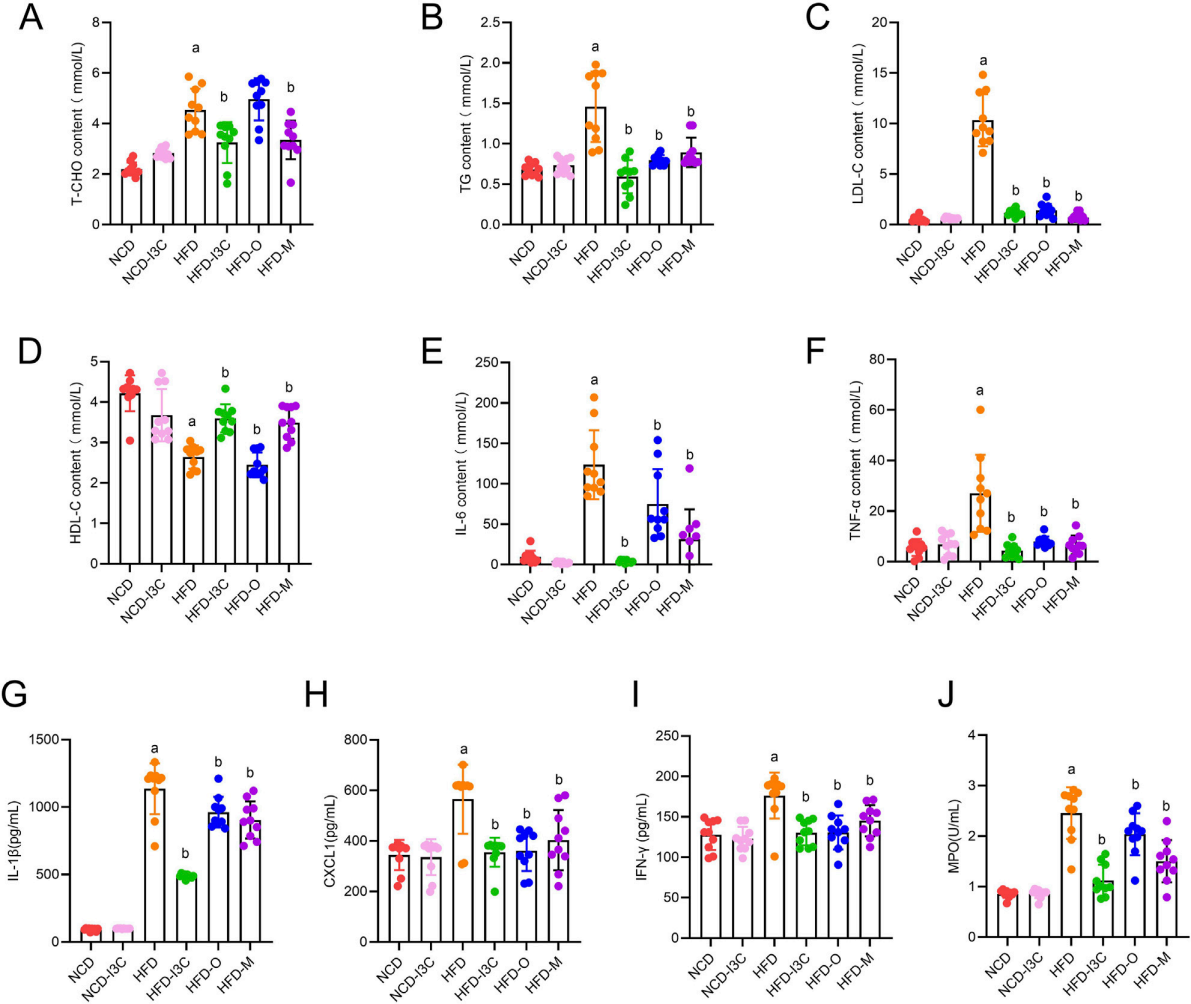
Supplementing I3C (40 mg/kg) to improve blood lipids and systemic inflammation in HFD mice. **(A)** T-CHO concentration; **(B)** TG concentration; **(C)** LDL-C concentration; **(D)** HDL-C concentration; **(E)** IL-6 concentration; **(F)** TNF-α concentration; **(G)** IL-1β concentration; **(H)** CXCL1concentration; **(I)** IFN-γ concentration; **(J)** MPO concentration. ^a^ *P* < 0.05, indicating comparison with NCD; ^b^ *P* < 0.05, indicating comparison with HFD. Data were expressed as mean ± SEM. (*n* = 10).

TG, TC, LDL-C, and HDL-C levels in NCD-fed mice displayed no significant changes after I3C treatment. Relative to the NCD mice, noticeable elevations were detected in TG, TC, and LDL-C levels with a remarkable reduction in HDL-C in the HFD-exposed mice (*P* < 0.05 or *P* < 0.01). After treatment with I3C, Orlistat, or Metformin, the TG, TC, and LDL-C levels in HFD-fed mice were markedly reduced, while that of HDL-C was noticeably raised (*P *< 0.05 or *P *< 0.01) (Figures 4A–D). Moreover, the regulatory impact of I3C on blood lipid levels was superior to the positive control Orlistat and almost equal to Metformin. Therefore, the intake of I3C could ameliorate blood lipid abnormalities triggered by HFD in mice and modulate the lipid metabolism disturbance of obese mice.

The IL-6, TNF-α, IL-1β, CXCL1, IFN-γ, and MPO levels in mouse plasma had no noticeable differences between NCD and NCD-I3C groups. The plasma levels of the aforementioned proteins were sharply upregulated in HFD-fed mice (*P *< 0.01). I3C, Orlistat, or Metformin treatment resulted in significant reductions in the plasma levels of these inflammatory proteins in HFD-fed mice (Figures 4E–J). The anti-inflammation action of I3C was superior to positive controls Orlistat and Metformin. It was illustrated that I3C could relieve HFD-elicited systemic inflammation.

### 3.4 Effects of I3C on HFD-evoked hepatic steatosis in mice

**FIGURE 5:**
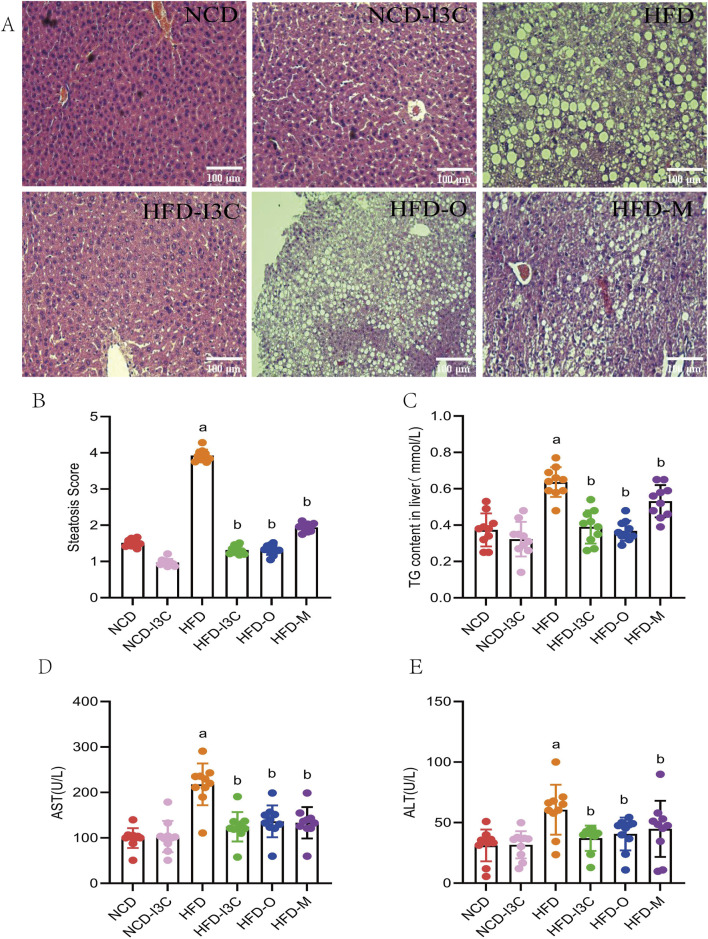
The treatment effect of I3C (40 mg/kg) on HFD induced liver steatosis in mice. **(A)** HE staining of liver tissue sections; **(B)** Steatosis Score; **(C)** TG concentrations in liver; **(D)** AST concentration; **(E)** ALT concentration. ^a^ *P* < 0.05, indicating comparison with NCD; ^b^ *P* < 0.05, indicating comparison with HFD. Data were expressed as mean ± SEM. (*n* = 10).

The pathological results showed good overall structure and tight arrangement of hepatocytes with uniform cytoplasm but no edema in the NCD and NCD-I3C groups, and a small amount of lipid droplets were visible. The HFD group exhibited slight swelling in the cytoplasm of hepatocytes, swelling of some organelles, accumulation of massive lipid droplets, and severe inflammatory cell infiltration. I3C treatment reversed the hepatic steatosis induced by HFD. In the HFD-I3C groups, we observed uniform cytoplasm of hepatocytes without swelling, slight swelling of organelles, and a small number of lipid droplets without obvious fusion. The results of the HFD-O group, HFD-M group, and HFD-I3C group were consistent (Figure 5A). To sum up, long-term intake of HFD will significantly damage the integrity of mouse liver tissue structure and promote the generation of massive lipid droplets in mice, and intragastric administration of I3C can significantly reduce lipid droplets, which may be the primary reason for lipid reduction.

No significant differences existed in histological scores, TC content in liver, AST, and ALT levels between the NCD and NCD-I3C groups. The aforesaid indicators markedly increased in the HFD-fed mice (*P *< 0.05 or *P *< 0.01). Additional I3C, Orlistat, or Metformin sharply downregulated the above indicators in the HFD-fed mice (*P *< 0.05 or *P *< 0.01) (Figures 5B–E). Relative to the positive controls (Orlistat and Metformin), I3C displayed a higher anti-inflammatory activity. These data together demonstrated that I3C could alleviate hepatic steatosis resulting from HFD.

### 3.5 Impacts of I3C on intestinal mucosal barrier in HFD-fed mice

**FIGURE 6:**
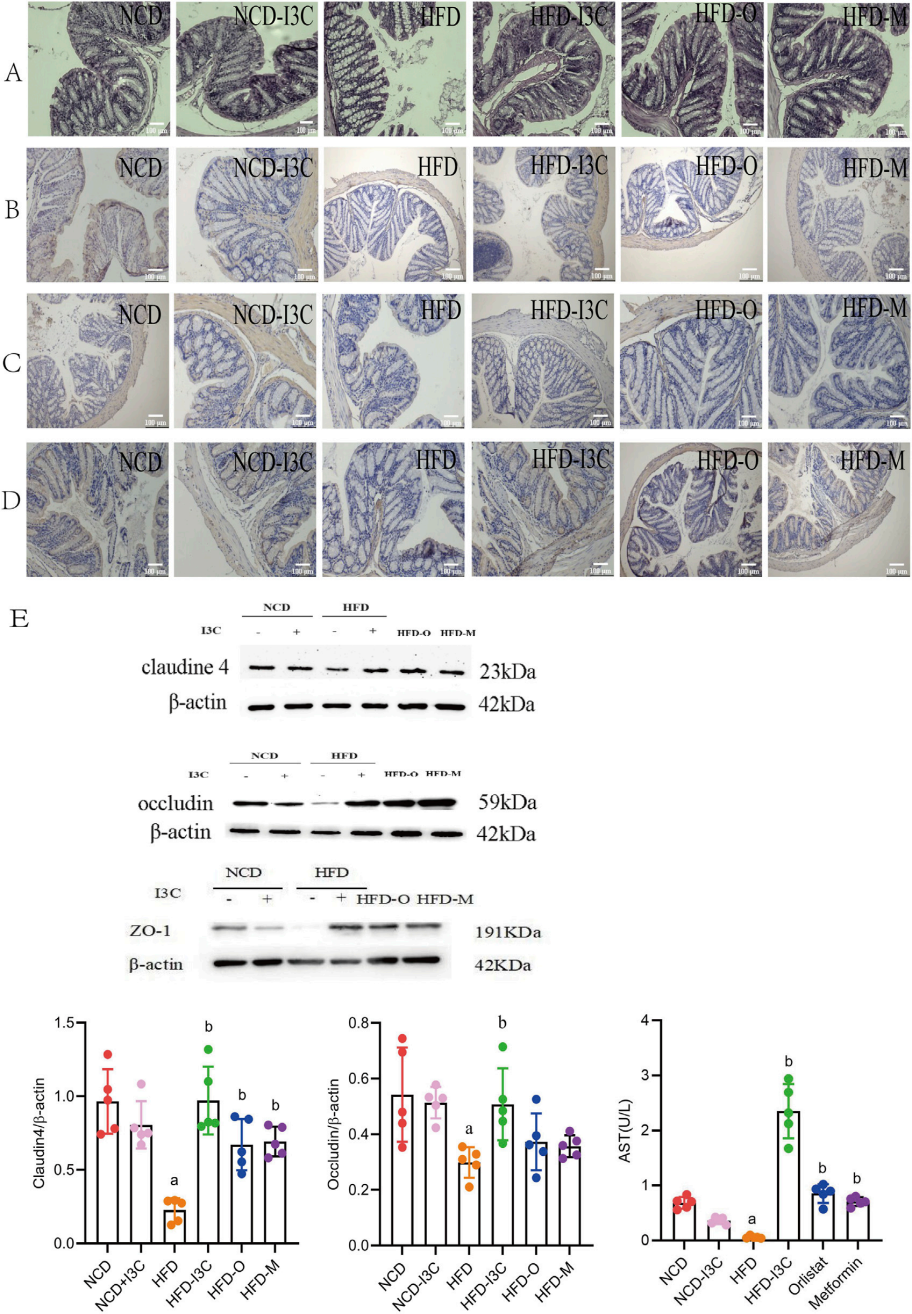
Supplementing I3C (40 mg/kg) to prevent HFD induced intestinal mucosal damage and inflammation in mice. **(A)** HE staining of colon; (*n* = 10) **(B)** Immunohistochemistry detection of Claudin 4 protein level; (*n* = 10) **(C)** Immunohistochemistry detection of Occludin protein level; (*n* = 10) **(D)** Immunohistochemistry detection of ZO-1; (*n* = 10) **(E)** Western Blotting detection of Claudin4, Occludin and ZO-1 protein levels in the colon. (*n* = 5) ^a^ *P* < 0.05, indicating comparison with NCD; ^b^ *P* < 0.05, indicating comparison with HFD. Data were expressed as mean ± SEM.

According to the pathological analysis results of colon tissue sections, the NCD-I3C group displayed no significant reduction in the colon length, with intact structure of intestinal mucosal epithelium and no infiltration of inflammatory cells, as compared to the NCD group. In comparison with the NCD-fed mice, HFD-induced mice had significantly shortened colon length, incomplete intestinal mucosal epithelium, large area defects, significant reduction of goblet cells, and infiltration of massive inflammatory cells deep into the submucosa. I3C therapy reversed the colon injury induced by HFD. I3C treatment led to a significantly prolonged colon length, an increased number of goblet cells, reduced inflammatory cell infiltration, and intact intestinal mucosal epithelium in the HFD-fed mice. The results of the HFD-O group, HFD-M group, and HFD-I3C group were consistent (Figure 6A).

No significant changes were noted regarding the levels of Claudin4, Occludin, and ZO-1 proteins in the NCD-fed mice after I3C administration. These proteins were noticeably downregulated in the HFD-fed mice as compared to the NCD mice (*P* < 0.05 or *P* < 0.01). Furthermore, I3C, Orlistat, or Metformin administration markedly augmented the aforementioned protein levels in the HFD-fed mice (*P *< 0.05 or *P *< 0.01) (Figures 6B–F). In comparison with the positive controls (Orlistat and Metformin), I3C exhibited superior function to improve the intestinal mucosal barrier function of mice. To sum up, I3C could attenuate HFD-evoked intestinal barrier damage.

### 3.6 Influences of I3C on the diversity of gut microbiota in HFD-fed mice

This research collected 4,222,786 high-quality 16S rRNA V3-V4 region base sequences in total. After examination of quality and chimeras as well as elimination of low-quality base sequences, 17,059 ASVs were identified. The ASV numbers in the NCD, NCD-I3C, HFD, HFD-I3C, HFD-O, and HFD-M groups were 4,656, 1812, 4,528, 2,347, 1940, and 1778, respectively. It was suggested that long-term intake of HFD could reduce the diversity of gut microbiota, which could be further diminished upon I3C treatment (Supplementary Figures S1A–E).

The results of α-diversity exhibited noticeable decreases the in diversity of gut microbiota in the NCD-I3C and HFD groups than in the NCD group (*P *< 0.05 or *P *< 0.01) as well as more significant reductions in the HFD-I3C, HFD-O, and HFD-M groups than in the HFD group (*P *< 0.05 or *P *< 0.01) (Supplementary Figures S1F–I). Conclusively, long-term intake of I3C in healthy mice and obese mice could significantly reduce the diversity of gut microbiota.

For β-diversity, the results of principal coordinate analysis (PCoA) based on the Bray-Cruits distance matrix and NMDS analysis, the NCD, NCD-I3C, HFD-I3C, HFD-O, and HFD-M groups displayed significant separation of gut microbiota without intersection phenomenon. Among these groups, the HFD-I3C group showed the distribution of gut microbiota close to the NCD group. Accordingly, significant differences existed in microbiota composition between the HFD group and other groups (Supplementary Figures S1J–L). The obesity model was successfully established, and I3C could mediate the composition of gut microbiota.

### 3.7 Effects of I3C treatment on the compositions of gut microbiota in HFD-fed mice

**FIGURE 7:**
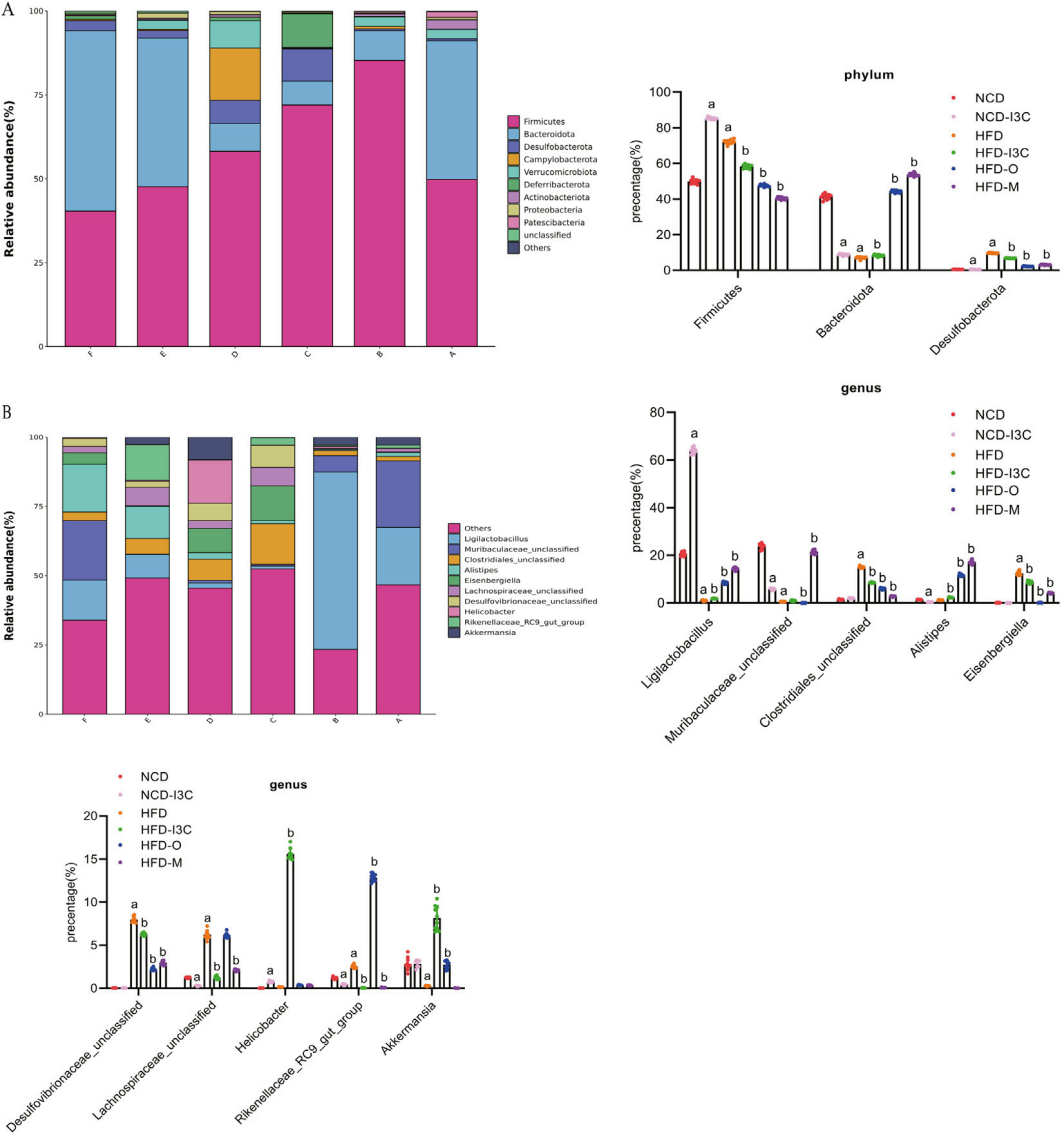
I3C (40 mg/kg) modulated the composition of the gut microbiota in different classification levels. **(A)** Phylum; **(B)** Genus. ^a^ *P* < 0.05, indicating comparison with NCD; ^b^ *P* < 0.05, indicating comparison with HFD. Data were expressed as mean ± SEM. (*n* = 10).

Herein, the experiment identified 25 phyla and 465 genera. The bacterial phyla in all groups were dominated by *Firmicutes*, *Bacteroidota*, and *Desulfobacterota*. The relative abundance of *Firmicutes* was 49.83%, 85.28%, 72.00%, 58.18%, 47.66%, and 40.38% in the NCD, NCD-I3C, HFD, HFD-I3C, HFD-O, and HFD-M groups, respectively. *Bacteroidota* accounted for 29.73%, 15.29%, 17.26%, 28.28%, and 30.10% while *Desulfobacterota* constituted 0.57%, 6.84%, 9.65%, 6.84%, 2.21%, and 3.04% in the NCD, NCD-I3C, HFD, HFD-I3C, HFD-O, and HFD-M groups, respectively (Figure 7A).

In contrast to the NCD group, the abundance of *Firmicutes*, abundance of *Desulfobacterota*, and *Firmicutes/Bacteroidota* ratio were dramatically raised (*P *< 0.01), while the abundance of *Bacteroidota* markedly decreased (*P *< 0.01) in the NCD-I3C and HFD groups. The *Firmicutes* abundance, *Desulfobacterota* abundance, and *Firmicutes/Bacteroidota* ratios sharply decreased (*P *< 0.01) but the *Bacteroidota* abundance significantly increased (*P *< 0.01) in the HFD-I3C, HFD-O, and HFD-M groups as compared to the HFD group.

The Circos plot was adopted to analyze the distribution of dominant gut microbiota in different taxa, the top 10 species ranked at the genus level and their relative abundance were identified as follows: *Ligilactobacillus* (18.44%), *Muribaculaceae_unclassified* (8.79%), *Clostridiales_unclassified* (6.00%), *Alistipes* (5.71%), *Eisenbergiella* (4.28%), *Desulfovibrionaceae_unclassified* (3.25%)*, Lachnospiraceae_unclassified* (2.88%)*, Helicobacter* (2.88%)*, Rikenellaceae_RC9_gut_group* (2.86%), and *Akkermansia* (2.80%) (Figure 7B).

**FIGURE 8:**
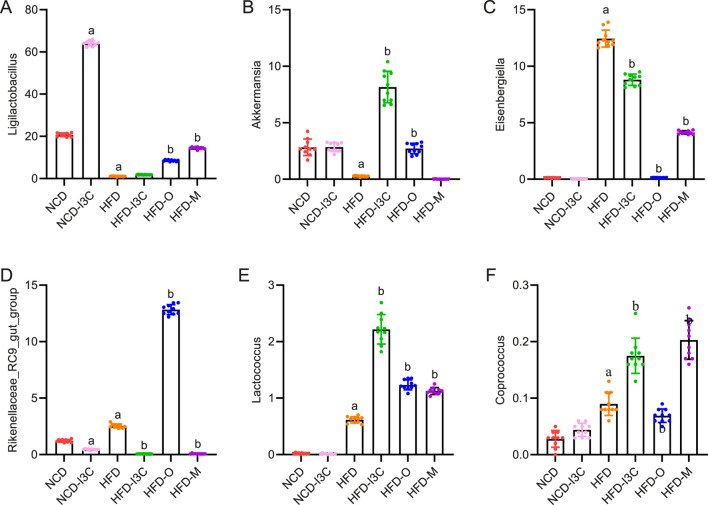
Effects of I3C (40 mg/kg) on intestinal flora abundance in obese mice at Phylum level. At Phylum level: **(A)** *Ligilactobacillus*; **(B)** *Akkermansia*; **(C)** *Eisenbergiella*; **(D)** *Rikenellaceae_RC9_gut_group*; **(E)** *Lactococcus*; **(F)** *Coprococcus*; ^a^ *P* < 0.05, indicating comparison with NCD; ^b^ *P* < 0.05, indicating comparison with HFD.

NCD-I3C and HFD groups displayed remarkably higher abundance of *Eisenbergiella* and *Rikenellaceae_RC9_gut_group* (*P *< 0.01) but notably lower abundance of *Akkermansia* (*P *< 0.01) versus the NCD group. After I3C, Orlistat, or Metformin treatment, the relative abundance of *Eisenbergiella* and *Rikenellaceae_RC9_gut_group* was notably reduced (*P* < 0.01), and that of *Akkermansia* markedly raised (*P *< 0.01) in HFD-fed mice (Figures 8A–D).

Significant reductions were noted concerning the abundance of microbiota related to SCFA production (*Lactococcus* and *Coprococcus*) in the NCD-I3C and HFD groups than in the NCD group (*P *< 0.01). Reversely, *Lactococcus* and *Coprococcus* showed higher abundance in the HFD-I3C, HFD-O, and HFD-M groups than in the HFD group (*P *< 0.01) (Figures 8E,F).

**FIGURE 9:**
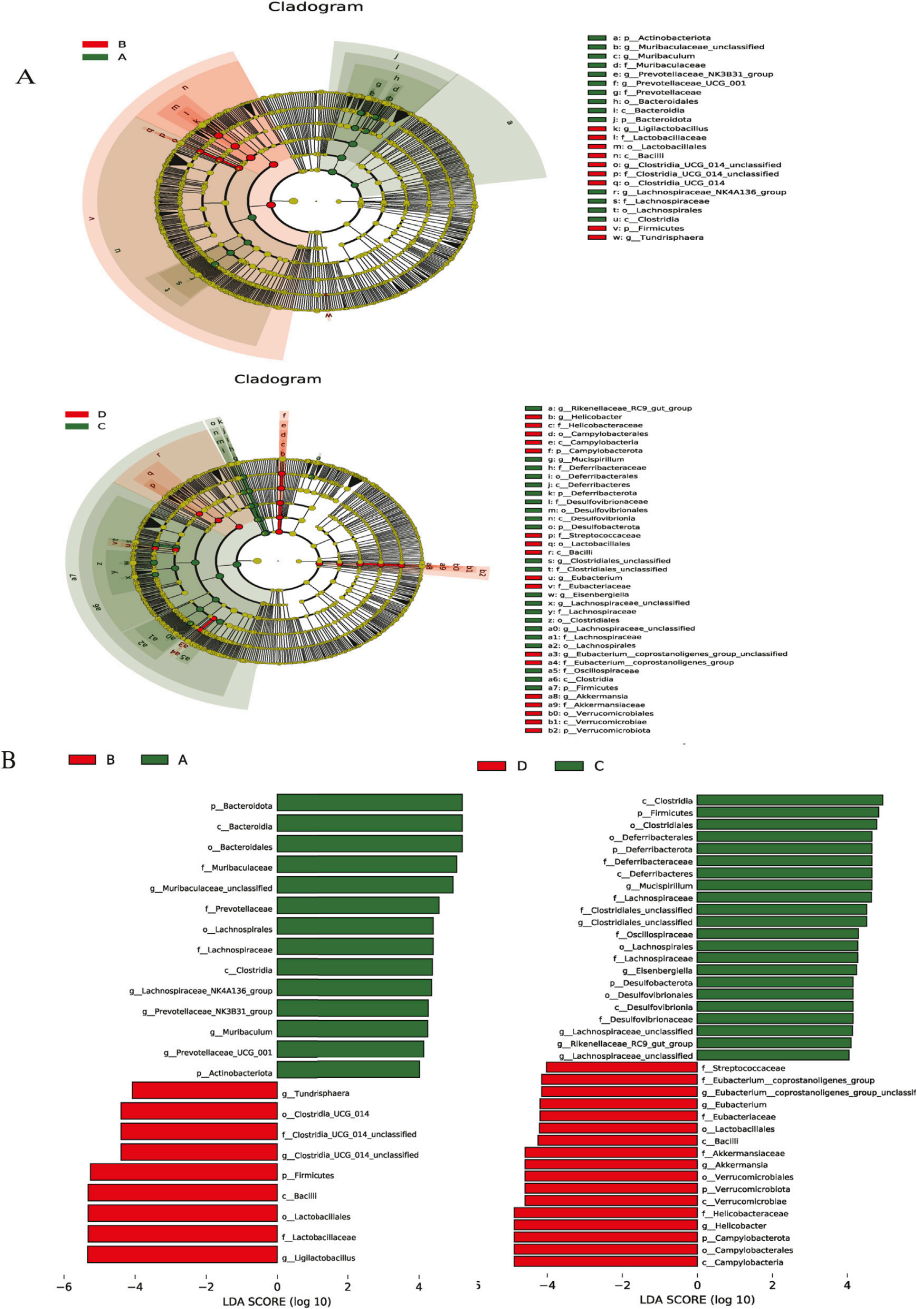
Key phylotypes of the gut microbiota that responded to I3C (40 mg/kg) supplementation in the NCD or HFD mice. **(A)** Cladogram generated from LEfSe analysis showing the relationship between taxon (the levels represent, from the inner to outer rings, phylum, class, order, family, and genus); **(B)** linear discriminant analysis (LDA) scores derived from LEfSe analysis, showing the biomarker taxa LDA score of >4 (the length of the bar represents the LDA score). **(A)** NCD group, **(B)** NCD-I3C group, **(C)** HFD group, **(D)** HFD-I3C group.

LEfSe analysis with a logarithmic LDA score threshold setting as 4 was employed for the identification of discriminative features. The NCD group was featured by relatively high abundance of *Muribaculaceae_unclassified*, *Lachnospiraceae_NK4A136_group*, and *Prevotellaceae_NK3B31_group*. The NCD-I3C group was characterized by a relatively high abundance of *Ligilactobacillus* and *Clostridiales_UCG_014_unclassified*. The HFD group exhibited a sharp elevation in the abundance of *Mucispirillum*, *Clostridiales_unclassified*, and *Eisenbergiella*. The HFD-I3C group showed a notably elevated abundance of *Helicobacter*, *Akkermansia*, and *Eubacterium* (Figure 9).

### 3.8 Correlations of gut microbiota changes with blood glucose, blood lipids, and inflammatory cytokines

**FIGURE 10:**
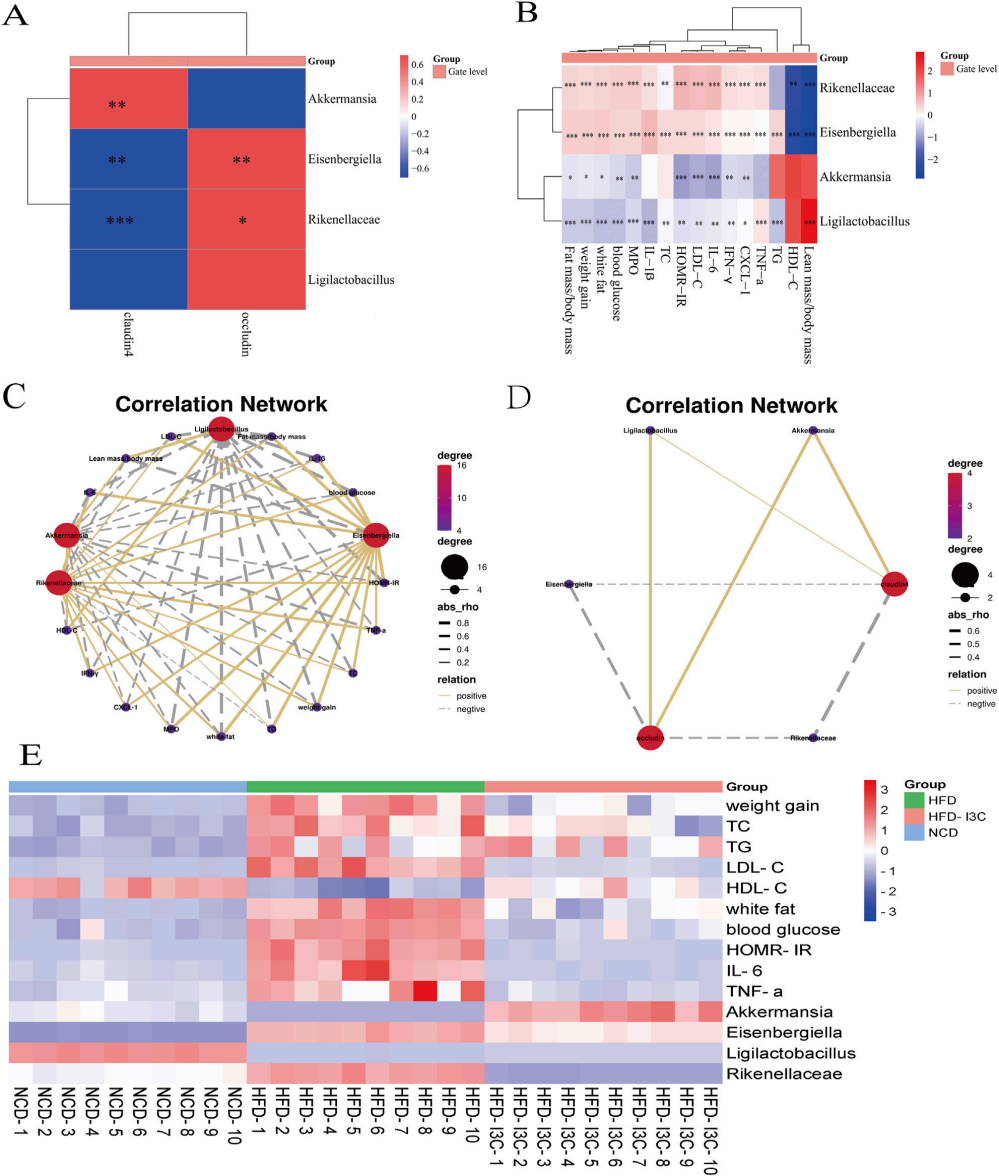
The Correlation between the changes of gut microbiota and the levels of blood glucose, blood lipids and inflammatory factors at the genus level. **(A)** Correlation analysis of bacteria with intestinal barrier protein; **(B)** Correlation analysis between bacteria and obesity factor; **(C)** Correlation network between bacteria and obesity factor; **(D)** Correlation network between bacteria and intestinal barrier protein; **(E)** Heatmap based on the levels of obesity-related indicators and relative abundance of key intestinal bacteria in NCD, HFD, HFD-I3C mice. ^*^ *P* < 0.05, ^**^ *P* < 0.01, ^***^ *P* < 0.001, ^****^ *P* < 0.0001. Data were expressed as mean ± SEM. (*n* = 10).

*Akkermansia* and *Ligilactobacillus* were reversely correlated with Fat mass/body mass, weight gain, and white fat but positively associated with Lean mass/body mass. Additionally, *Akkermansia* and *Ligilactobacillus* shared negative associations with TC, TG, HOMR-IR, blood glucose, and LDL-C but a positive correlation with HDL-C. Also, these two bacteria were positively linked to claudin 4 and occludin (Figure 10). It was indicated that the aforementioned two bacteria might exert anti-obesity effects by controlling lipid metabolism and restoring intestinal barrier function.

*Eisenbergiella* and *Rikenellaceae_RC9_gut_group* were positively related to Fat mass/body mass, weight gain, and white fat but negatively correlated with Lean mass/body mass. Meanwhile, *Eisenbergiella* and *Rikenellaceae_RC9_gut_group* were positively linked to TC, TG, HOMR-IR, blood glucose, and LDL-C but inversely linked to HDL-C. These two bacteria also displayed negative associations with claudin 4 and occludin. The above-mentioned two bacteria were, therefore, suggested to potentially induce obesity by increasing white fat content, disrupting lipid metabolism, and disrupting the intestinal barrier.

### 3.9 Significance of I3C in metabolic characteristic prediction of gut microbiota composition in HFD-fed mice

**FIGURE 11:**
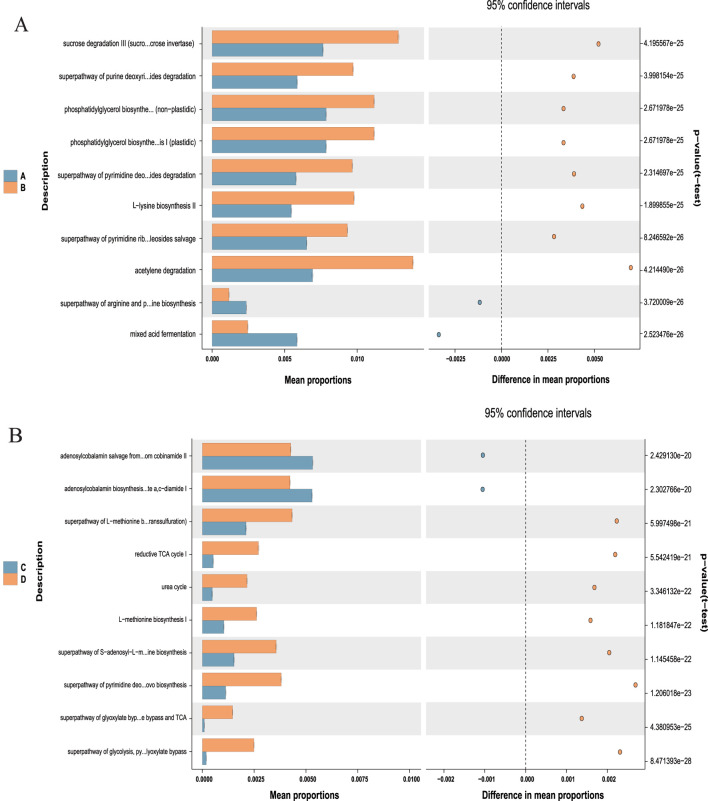
Predicted metabolic profile of the fecal microbiome after different treatments. **(A)** A vs. B is NCD vs. NCD-G; **(B)** C vs. D is HFD vs. HFD-G. 16S rRNA data were further analyzed as indicated by the PICRUSt. Statistical significance difference among treatment groups based on Welch’s *t*-test (*P* < 0.001). in STAMP. The colored circles represent 95% confidence intervals calculated using Welch’s inverted method.

Given the above-mentioned structural changes, we further analyzed the 16S rRNA data and performed PICRUSt functional predictions (Figure 11). On the strength of Welch’s *t*-test (*P *< 0.001), the metabolic characteristics of gut microbiota were analyzed. Compared with the NCD group, the HFD group exhibited a noticeable downregulation in the expression of 10 KEGG pathways, which was significantly upregulated after I3C administration: adenosine ribonucleotides *de novo* biosynthesis, aromatic biogenic amine degradation (bacteria), anhydromuropeptides recycling, adenosine deoxyribonucleotides *de novo* biosynthesis II, acetylene degradation, 4-deoxy-L-threo-hex-4-enopyranuronate degradation, 4-aminobutanoate degradation V, 2-methylcitrate cycle I, 2-methylcitrate cycle II, 1,4-dihydroxy-2-naphthoate biosynthesis I. In contrast to the NCD group, the HFD group showed significant upregulation of 5 KEGG pathways, including adenosylcobalamin salvage from cobinamide I, adenosylcobalamin salvage from cobinamide II, adenosylcobalamin biosynthesis from cobyrinate a,c-diamide I, acetyl-CoA fermentation to butanoate II, 1,4-dihydroxy-6-naphthoate biosynthesis I. These pathways were significantly downregulated after I3C administration.

### 3.10 Impacts of I3C on the serum metabolite balance in HFD-fed mice

**FIGURE 12:**
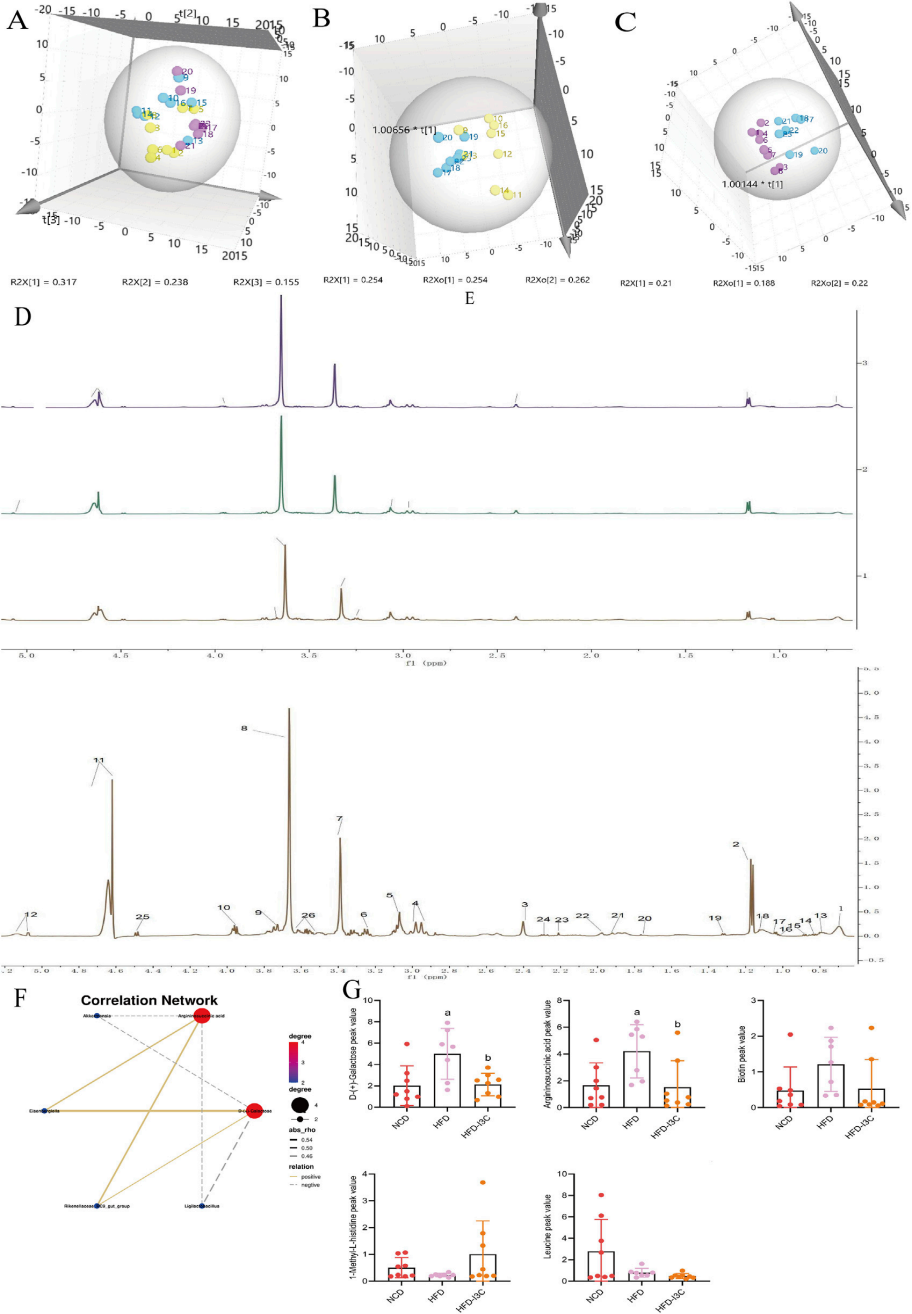
I3C (40 mg/kg) treatment effect on serum metabolism (n = 7). **(A)** Scores plot of PCA analysis of all samples; **(B)** Scores plot of PLS-DA of HFD vs. NCD; **(C)** Scores plot of PLS-DA of HFD vs. HFD-I3C; **(D)** Serum H-NMR spectra of HFD, NCD and HFD-I3Cgroup; **(E)** Amplification of common peak of serum H-NMR spectrum in each group; Numbers represent the peaks of metabolites: 1. VLDL; 2. Lactic acid; 3. Methylamine; 4 Glutathione; 5. Creatine; 6. Tyramine; 7.1-Methyl-L-histidine; 8. Glycerophosphocholine; 9. Glucose; 10. Argininosuccinic acid; 11. D-Galactose; 12. Biotin; 13. L-Isoleucine; 14. Leucine; 15. Valine; 16.3-hydroxybutyric acid; 17. Lipids; 18. LDL; 19. Alanine; 20. Acetate; 21. Glycoprotein; 22. Methionine; 23. Pyruvic acid; 24. Carnitine; 25. Lactose; 26. N-Acetyl-D-mannosamine; **(F)** Correlations network between gut microbiota and serum metabolites; **(G)** I3C treatment effect on contentof differential metabolite. ^a^ *P* < 0.05, indicating comparison with NCD; ^b^ *P* < 0.05, indicating comparison with HFD. Data were expressed as mean ± SEM. (*n* = 7).

PCA data yielded 5 principal components. The parameters R^2^X (cum) and R^2^Y (cum) in the NCD and HFD groups were 0.543 and 0.851, respectively. *R* ^2^ was relatively large, indicating good model fit accuracy. Pattern recognition analysis was carried out pairwise on different groups to reflect differences in samples and overall metabolic differences. A significant separation among the HFD, NCD, and HFD-I3C groups was visible in a PCA score chart, suggestive of alterations in serum metabolites triggered by HFD. These metabolites were also changed after I3C administration (Figures 12A–C). The metabolic characteristics were compared among these three groups through H-NMR metabolomics analysis (Figures 12D,E). Relative to the NCD mice, the HFD mice displayed 9 differentially expressed metabolites (Table 1). The HFD and HFD-I3C groups exhibited 6 differentially expressed metabolites (Table 2). The intersection metabolites among the aforementioned three groups were argininosuccinic acid and galactose (Figure 12G).

**TABLE 1:** Differentially expressed metabolites in the NCD and HFD group.

Metabolites	Chemical shift (ppm)	Trend	VIP	P(Corr)
D-(+)-Galactose	4.63(d)3.64(m)	↑	31.014	0.778
Argininosuccinic acid	3.96(d)3.64(m)	↑	24.138	0.684
Lactose	4.49(d)3.66(m)	↓	23.706	−0.522
Glycerophosphocholine	3.67(s)	↑	12.276	−0.481
L-Isoleucine	0.77(d)3.65(m)	↑	7.707	−0.45
Biotin	5.10(d)3.35(m)	↑	6.692	0.486
Glucose	3.74(d)3.40(m)	↓	3.379	−0.472
1-Methyl-L-histidine	3.39(s)3.34(m)	↓	11.746	0.717
leucine	0.85(d)3.33(m)	↓	1.364	0.436

**TABLE 2:** Differentially expressed metabolites in the HFD and HFD-I3C group.

Metabolites	Chemical shift (ppm)	Trend	VIP	P(Corr)
D-(+)-Galactose	4.63(d)3.64(m)	↓	31.273	0.73
Argininosuccinic acid	3.96(d)3.64(m)	↓	28.315	0.656
Biotin	5.10(d)3.35(m)	↓	8.184	0.464
leucine	0.85(d)3.33(m)	↓	4.435	−0.371
1-Methyl-L-histidine	3.39(s)3.34(m)	↑	13.166	0.694
N-Acetyl-D-mannosamine	3.62(m)	↓	4.923	−0.439

### 3.11 I3C Supplementation ameliorates the metabolic disorders in HFD-fed mice by modulating gut microbiota

We analyzed the potential correlation between gut microbiota abundance (genus level) and serum metabolites (Figure 12F; Table 3) and found a strong and broad correlation between bacterial species and serum metabolites. *Akkermansia*, *Eisenbergiella*, *Ligilactobacillus*, and *Rikenellaceae_RC9_gut_group*, were correlated with the changes in metabolites Argininosuccinic acid and D-(+)-Galactose in serum.

**TABLE 3:** Correlations between gut microbiota and serum metabolites.

Phylum	Genus	Metabolites	R	P
*Verrucomicrobiota*	*Akkermansia*	Argininosuccinic acid	−0.4299	0.0406
		D-(+)-Galactose	−0.4497	0.0313
*Firmicutes*	*Eisenbergiella*	Argininosuccinic acid	0.5094	0.0130
		D-(+)-Galactose	0.5796	0.0037
*Firmicutes*	*Ligilactobacillus*	Argininosuccinic acid	−0.4506	0.0309
		D-(+)-Galactose	−0.4960	0.0161
*Bacteroidota*	*Rikenellaceae_RC9_gut_group*	Argininosuccinic acid	0.4585	0.0278
		D-(+)-Galactose	0.5287	0.0095

## 4 Discussion

The pathogenesis of obesity involves gut microbiota disturbance and low-grade inflammation in the body (Cani et al., 2007; Honda and Littman, 2012). Hence, the modulation of gut microbiota and repair of the intestinal mucosal barrier may be of great significance in treating obesity. A vegetable-rich diet can prevent obesity. Dietary fiber is beneficial for obesity, and indoles in vegetables also have great potential for the treatment of obesity (Bindels et al., 2015). I3C exhibits positive roles in repairing the intestinal mucosal barrier, preventing enteritis and colon cancer, and preventing and treating obesity. This study adopted I3C administration for treatment in the mouse model of obesity and explored the mechanism of I3C in treating obesity from the perspectives of gut microbiota, intestinal mucosal barrier, metabolism, etc.

Fat and environmental dysregulation of the body driven by fat can lead to low-grade systemic inflammation and intestinal inflammation (Zou et al., 2018). The levels of TNF-α, IL-6, IL-1β, INF-γ, CXCL1, and MPO were upregulated in the plasma of HFD mice. IL-6 is secreted in brown adipocytes, making these cells unable to regulate the metabolism of substances such as glucose and decompose fat, ultimately leading to obesity (Al-Sadi and Ma, 2007). IL-1β stimulates insulin secretion through central muscarine signaling, which leads to increased food intake; it also inhibits fat breakdown to cause fat accumulation in the body, resulting in obesity (Tilg and Kaser, 2011). Pro-inflammatory cytokines (such as TNF-α, IL-6, and INF-γ) are mainly sourced from macrophages. The secretion of multiple pro-inflammatory cytokines and chemokines (such as CXCL1) gives rise to the accumulation of M1 macrophages in adipose tissues, leading to insulin resistance and obesity (Barra et al., 2010; McGillicuddy et al., 2011; Chang et al., 2012; Kawano et al., 2016). Our research indicated that I3C treatment markedly downregulated the levels of TNF-α, IL-6, IL-1β, INF-γ, CXCL1, and MPO in the plasma of HFD-fed mice, reduced local and systemic inflammation in the intestine. Additionally, I3C treatment could improve insulin sensitivity, glucose tolerance, and insulin resistance and also regulate lipid metabolism disorders in HFD-fed mice. I3C might exert a therapeutic effect against obesity by reducing intestinal and systemic inflammation and relieving insulin resistance.

The intestinal barrier is the barrier between the host and the environment that protects the host from external toxins, dietary antigens, pathogenic bacteria, and other potentially harmful substances (Kirpich et al., 2012). The intestinal barrier is composed of tight junctions (TJs) and adherens junctions (AJs) (Kirpich et al., 2012). TJs are polymer complexes composed of membrane proteins like occludin and claudin4 (Rao et al., 2002). Its intracellular domain binds to “bridging” proteins such as ZO-1 (Usami et al., 2003), directly anchoring TJs to F-actin to form sealed lumina, thereby selectively allowing the penetration of nutrients into the cells and inhibiting unnecessary penetration of environmental toxins, luminal antigens, and bacteria (Usami et al., 2001). Fat downregulates the expression of occludin, claudin1, and claudin4 proteins, which allows the penetration of luminal substances into the lamina propria and reduces the tightness of TJs (Günzel and Yu, 2013). This study validated the downregulation of intestinal TJ proteins (claudin4, occludin, and ZO-1) in HFD-fed mice and infiltration of macrophages in mouse liver and colon epithelium, suggesting that HFD disrupted the intestinal barrier, induced systemic inflammation, consistent with literature reports. However, I3C administration significantly augmented the levels of these proteins in HFD-exposed mice, restored the intestinal barrier, and protected the host from toxins, contributing to an anti-obesity effect.

This article explores the relationship between gut microbiome dysbiosis and obesity as well as type 2 diabetes. The alpha diversity of obese individuals is significantly lower than that of healthy individuals. At the phylum level, the ratio of *Firmicutes* to *Bacteroidetes* in obese individuals is relatively low (Voruganti, 2023). At the genus level, the relative abundance of *Ruminococcus* is higher among obese individuals, while the relative abundances of *Prevotella*, *Akkermansia*, and *Methanobacteriales* are lower (Belda et al., 2022). Type 2 diabetes patients have a significantly lower total number of *Bifidobacteria* and *Lactobacilli* in their guts, *Bacteroidetes* and *Proteobacteria* increase, *Firmicutes* decreases, and the ratio of *Bacteroidetes* to *Firmicutes* rises (Tsai et al., 2014; Pinart et al., 2021). The proportion of the *Betaproteobacteria* class is significantly higher in diabetes patients and is positively correlated with the increase in blood glucose concentration (Dabke et al., 2019). The gut microbiome of type 2 diabetic individuals is also correlated with a decrease in certain butyrate-producing bacteria. The gut microbiome influences host metabolism by regulating short-chain fatty acids, bile acids, branched-chain amino acids, and other metabolic products (Liu et al., 2022). This has an impact on host glucose regulation and insulin sensitivity, thereby modulating host metabolism (Canfora et al., 2019). After dysbiosis occurs, the dominant bacterial community with polysaccharide metabolism genes can decompose the carbohydrates in the host’s body, store fat, and thereby contribute to the occurrence of obesity and insulin resistance (Malesza et al., 2021). Discussing the relationship between the gut microbiome and obesity as well as type 2 diabetes can lead to the formulation of more effective prevention and treatment strategies. By adjusting the dietary structure, increasing dietary fiber intake, restoring the diversity of intestinal microflora, and facilitating the generation of beneficial metabolites, it is possible to enhance the health levels of obese and diabetic patients and prevent the occurrence of their complications.

This study unveiled that I3C could restore the gut microbiota structure damaged by HFD and facilitate the growth of certain specific bacteria in HFD-fed and NCD-fed mice. It has been suggested that the decrease in the diversity of gut microbiota of obese patients is related to obesity (Gou et al., 2023). Through the changes in the number of ASVs as well as Observed species, Shannon index, Simpson index, and Chao index of gut microbiota, the present study demonstrated that HFD reduced the diversity of gut microbiota, and positive drugs Orlistat and Metformin markedly reduced that of gut microbiota, which was further reduced by I3C administration. PCOA analysis showed a significant separation between the HFD-I3C and HFD groups. It was inferred from the diversity of gut microbiota that I3C reshaped the structure of gut microbiota (Supplementary Figure S1). The BUGbase analysis results showed a decreased abundance of potential pathogenic bacteria after I3C supplementation in the HFD-fed mice. I3C administration may inhibit the growth of pathogenic bacteria, responsible for its role in reducing the diversity of gut microbiota (Supplementary Figure S2). In this experiment, I3C successfully reversed the HFD-caused increase of *Firmicutes/Bacteroidota* ratio.

*Akkermansia* represents the representative genus of *Verrucomicrobia* (Zhang et al., 2011). *Akkermansia* decreases intestinal permeability in obese mice by reducing the plasma LPS levels (Hasani et al., 2021), enhances glucose tolerance, and alleviates systemic inflammation in obese mice *via* stimulating Foxp3^+^ regulatory T cells (Rodrigues et al., 2022). This experiment confirmed that I3C treatment in HFD-fed mice increased the abundance of *Verrucomicrobia* at the phylum level and that of *Akkermansia* at the genus level. Correlation analyses elucidated that *Akkermansia* levels were inversely correlated with TC, blood glucose, insulin, and fat content, but positively linked to claudin 4 and occludin, indicating that I3C could ameliorate low-grade inflammation and insulin resistance in mice by increasing the abundance of *Akkermansia* in *Verrucomicrobia*.

*Ligilactobacillus* levels. It has been proven that *Ligilactobacillus* can improve intestinal barrier function, release intestinal incretin hormone GLP-1, and reverse metabolic disorders (Liu et al., 2020). In this study, *Ligilactobacillus* decreased notably in HFD-fed mice, while its content in the NCD-I3C group was significantly higher than that in the NCD and HFD groups. This study also suggested a negative correlation between *Ligilactobacillus* and obesity parameters, consistent with the literature. I3C intervention could markedly increase *Ligilactobacillus* in the NCD-exposed mice and slightly increase *Ligilactobacillus* in the HFD-exposed mice, exerting an anti-obesity effect.

*Eisenbergiella* may be associated with the initiation of obesity and inflammatory diseases in females (Jang et al., 2019). Individuals who consume massive saturated fatty acids and a low-dietary fiber diet exhibit an increase in the abundance of *Eisenbergiella* (Bailén et al., 2020). This study revealed an elevated abundance of *Eisenbergiella* in the HFD group as well as positive correlations of *Eisenbergiella* with obesity-associated parameters but a negative correlation with HDL-C. I3C intervention remarkably reduced the increase in the abundance of *Eisenbergiella* caused by HFD, exerting its anti-obesity effects. Ferrer et al. found that the abundance of *Rikenellaceae* is relatively high in obese individuals, suggesting a correlation between *Rikenellaceae* and obesity (Ferrer et al., 2013). This experiment also confirmed that I3C intervention notably reduced the HFD-caused increase in the abundance of *Rikenellaceae_RC9_gut_group* and that *Rikenellaceae_RC9_gut_group* was positively linked to obesity-associated parameters such as white fat but inversely related to HDL-C. Altogether, the abundance of *Eisenbergiella* and *Rikenellaceae_RC9_gut_group* decreased, pro-inflammatory cytokines were downregulated, and intestinal mucosal barrier proteins were upregulated following I3C treatment, further supporting the correlations of *Eisenbergiella* and *Rikenellaceae_RC9_gut_group* with obesity.

SCFAs, final products of dietary ingredients fermented by gut microbiota (Canfora et al., 2015), have been unveiled to prevent metabolic endotoxemia, protect against inflammation, increase insulin sensitivity, enhance intestinal barrier function, and prevent diet-induced obesity (You et al., 2022). The present study demonstrated that I3C treatment led to significant elevations of SCFA-producing bacteria such as *Eubacterium*, *Lactococcus*, and *Coprococcus*. Also, I3C treatment could enhance insulin sensitivity, alleviate systemic inflammation, and strengthen intestinal barrier function. These results are consistent with the preventive function of SCFAs against obesity.

This study identified 9 types of dysregulated metabolites in the serum samples of obese mice, and I3C caused alterations in 6 kinds of metabolites. Argininosuccinic acid and galactose were the common dysregulated metabolites in the intersection. Some evidence shows that dietary supplementation of arginine can ameliorate obesity in rats with congenital obesity, rats with diet-induced diabetes, and patients with type 2 diabetes (Trevisson et al., 2007). The results of this study demonstrated that the serum levels of argininosuccinic acids were elevated in HFD-fed mice, which were reduced after I3C treatment. It was believed that I3C might promote the breakdown of argininosuccinic acids into arginine in the serum of obese mice, thereby alleviating the obesity symptoms in mice. Furthermore, an elevation of argininosuccinic acids gives rise to argininosuccinic aciduria (ASA) and also induces liver enlargement, elevated liver enzymes, and even severe liver fibrosis (Erez et al., 2011). Our study revealed that the levels of aspartate aminotransferase (AST) and alanine aminotransferase (ALT) increased in HFD-fed mice, but these levels were noticeably decreased after I3C treatment, which may be related to the decrease in the content of argininosuccinic acids induced by I3C.

Lactose is easily broken down into glucose and galactose by lactase in the intestine. Galactose is a component of the glycolipids that constitute the brain and nerve tissue (Azman and Zakaria, 2019). Long-term use of galactose can lead to metabolic transformation into aberrant metabolic pathways, leading to pathological changes extremely similar to natural aging (Briggs et al., 2017). In this study, the serum lactose content decreased and galactose increased in the HFD group, and I3C treatment reduced the serum galactose content in obese mice. Lactose is broken down into galactose and glucose to release energy and elevate blood glucose levels. The aforementioned findings illustrated that I3C potentially repressed lactose decomposition, thereby reducing blood glucose levels and energy release, contributing to the anti-obesity function.

At present, the metabolites argininosuccinic acids and galactose have not been linked to gut microbiota yet. The results of this study demonstrated that *Akkermansia* and *Ligilactobacillus* were inversely relevant to the contents of argininosuccinic acids and galactose. Reversely, *Eisenbergiella* and *Rikenellaceae_RC9_gut_group* were positively correlated with the contents of these two metabolites. It was inferred that I3C treatment could restore the metabolism of argininosuccinic acids and galactose by regulating the structure of gut microbiota, thereby exerting an anti-obesity effect.

## 5 Conclusion

I3C treatment reduces body weight, hepatic steatosis, and systemic inflammation of HFD-fed mice, ameliorates insulin resistance, and significantly augments the expression of Claudin4, Occludin, and ZO-1 to enhance intestinal barrier protein function. Additionally, it increases the enrichment of probiotics *Akkermansia* and *Ligilactobacillus* as well as SCFA-generating bacteria: *Eubacterium*, *Lactococcus*, and *Coprococcus*. I3C treatment also restrains the growth of *Eisenbergiella* and *Rikenellaceae_RC9_gut_group* and modulates the metabolism of argininosuccinic acids and galactose, showing its anti-obesity action. Our research supplies experimental evidence for the promise of I3C as a potential candidate therapeutic drug for obesity. This article discussed the molecular mechanism of I3C in the treatment of obesity, yet the cellular levels, signaling pathways, and adverse effects require further exploration. This work provides clear evidence for the pathogenesis of obesity and the treatment of I3C.

## Data Availability

The original contributions presented in the study are publicly available. This data can be found here: NCBI repository, accession number PRJNA1202745.

## Supplementary material

SUPPLEMENTARY FIGURE S1 Gut microbiota diversity analusis in response to different treatments. **(A)** Chao1 rarefaction curve of samples; **(B)** Shannon rarefaction curve of samples; **(C)** Species accum curve; **(D)** Rank abundance **(E)** Six experimental groups ASV Venn diagram; **(F)** Simpson index; **(G)** Chao index; **(H)** Observed otus index; **(I)** Shannon index; **(J)** Nonmetric multidimensional scaling (NMDS) score plot based on Bray Curtis; **(K)** Principal component analysis; **(L)** PCoA score plot based on Bray Curtis. ^*^ *P* < 0.05, ^**^ *P* < 0.01, ^***^ *P* < 0.001, ^****^ *P* < 0.0001. Data were expressed as mean ± SEM. (*n* = 10).

SUPPLEMENTARY FIGURE S2 Effects of I3C (40 mg/kg) on intestinal microbial phenotypes. **(A)** Aerobic; **(B)** Anaerobic; **(C)** Facultatively anaerobic; **(D)** Gram negative; **(E)** Gram positive; **(F)** Contains mobile elements; **(G)** Forms biofilms; **(H)** Potentially pathogentic; **(I)** Stress tolerant; **(J)** GO analysis of I3C treatment for obesity; **(K)** KEGG analysis of I3C treatment for obesity. Data were expressed as mean ± SEM. (n = 10).
