# Supplementary figures and images for: Modulation of gut microbiota, up-regulation of ZO-1, and promotion of metabolism as therapeutic mechanisms of indole-3-carbinol against obesity in mice
Source: Front Pharmacol. 2025 Jan 3;15:1499142. doi: 10.3389/fphar.2024.1499142 (PMC11739362; doi:10.3389/fphar.2024.1499142)

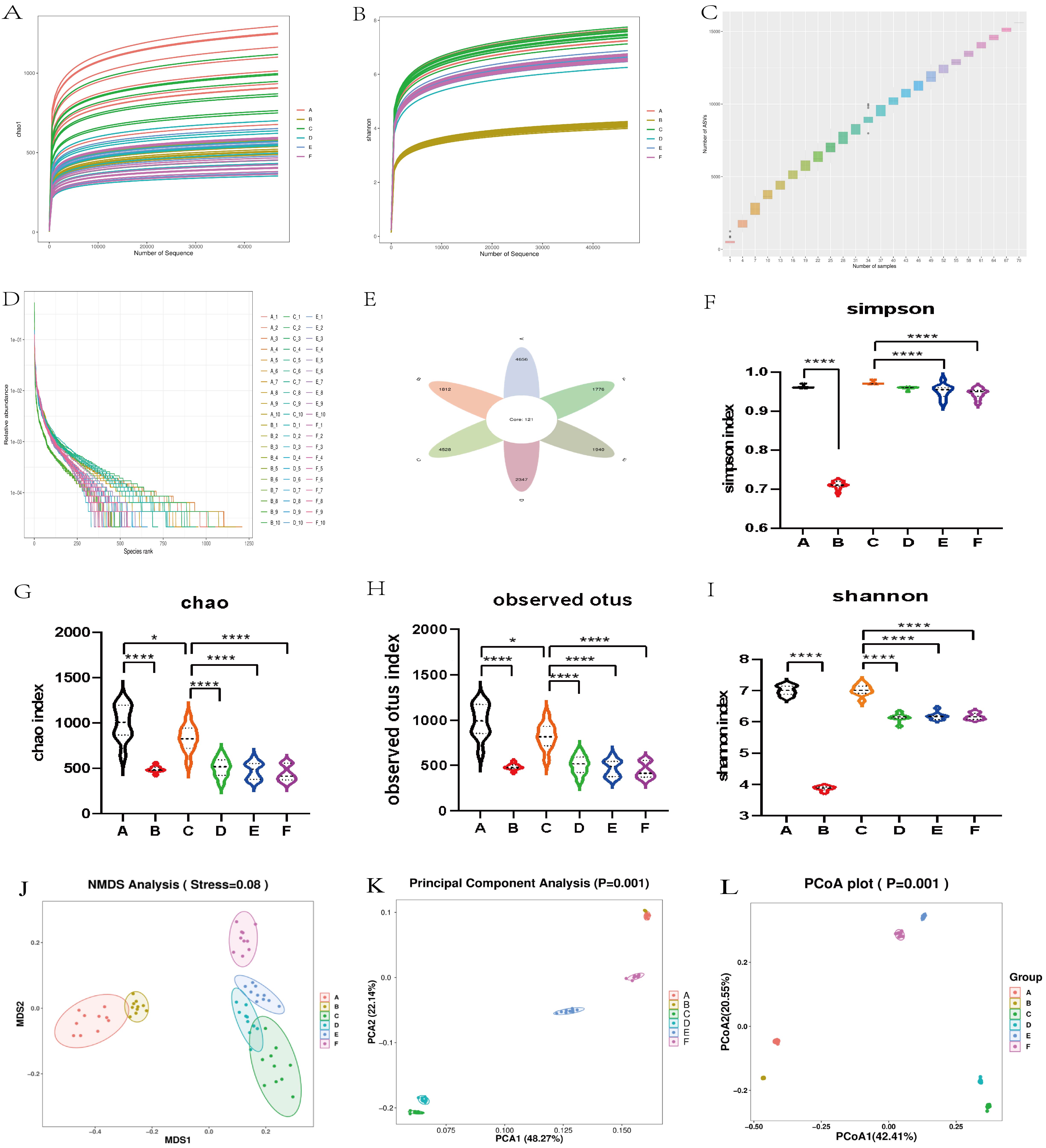

Supplement: Supplementary file 1 [file Image1.JPEG]

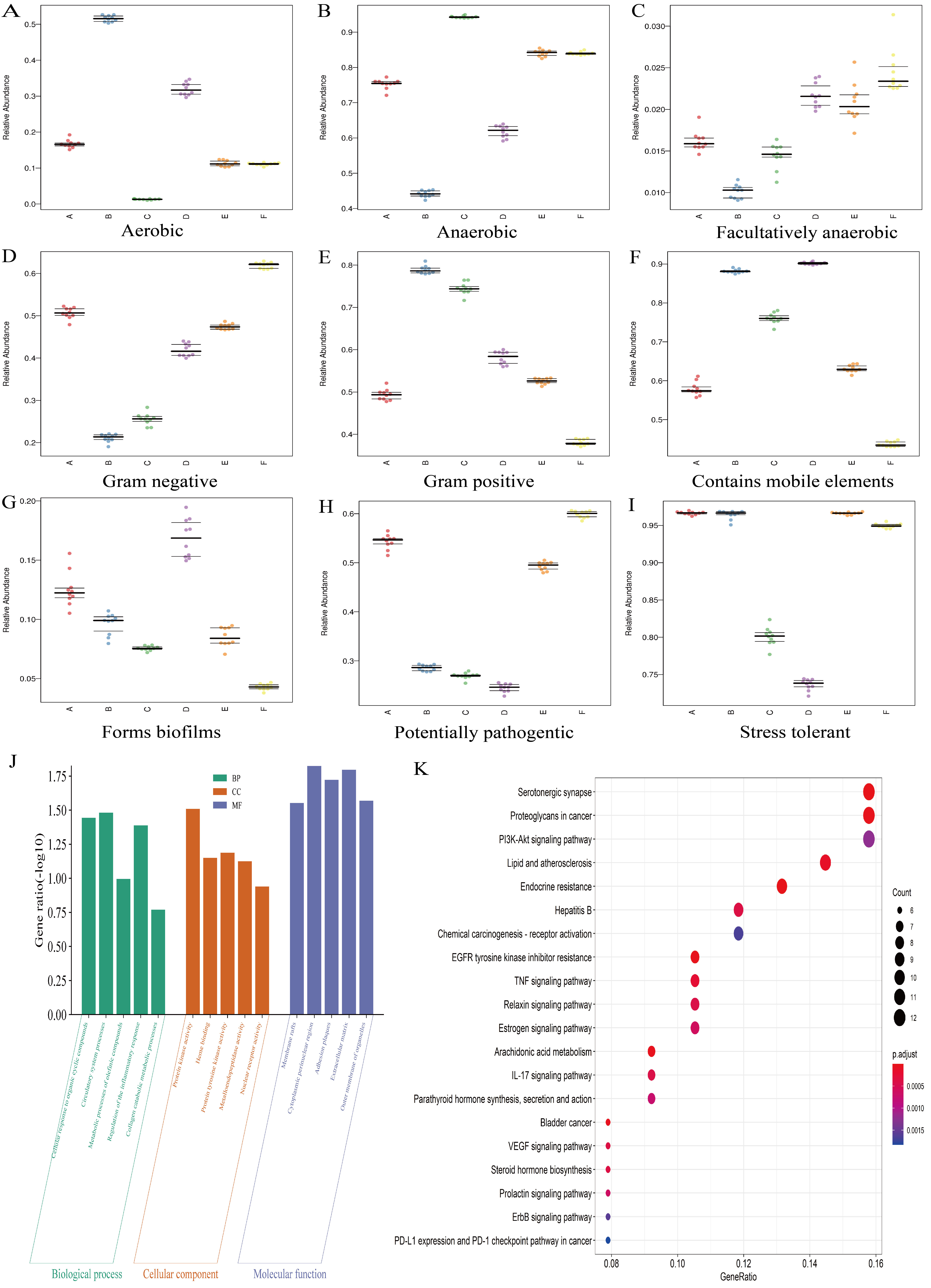

Supplement: Supplementary file 2 [file Image2.JPEG]
